# Genetic Analysis and Fingerprint Construction for *Isatis indigotica* Fort. Using SSR Markers

**DOI:** 10.3390/cimb47030146

**Published:** 2025-02-24

**Authors:** Xiangyu Xing, Haijun Xu, Yan Dong, Hanwen Cui, Mingrui Sun, Hong Wang, Yang Liu, Li Meng, Chunying Zheng

**Affiliations:** 1 Engineering Research Center of Agricultural Microbiology Technology, Ministry of Education & Heilongjiang Provincial Key Laboratory of Plant Genetic Engineering and Biological Fermentation Engineering for Cold Region & Key Laboratory of Molecular Biology, Heilongjiang University, Harbin 150080, China; 13325152141@163.com (X.X.); chw0319bb@163.com (H.C.); 18304528894@163.com (M.S.); 2021103@hlju.edu.cn (Y.L.); 2 Daqing Branch of Heilongjiang Academy of Sciences, Daqing 163319, China; nxynxhj@163.com (H.X.); dy0770@126.com (Y.D.); 3 Key Laboratory of Functional Inorganic Material Chemistry (Heilongjiang University), Ministry of Education, Harbin 150080, China; 2011028@hlju.edu.cn

**Keywords:** *Isatis indigotica* Fort., SSR markers, genetic identification, genetic diversity, fingerprinting

## Abstract

*Isatis indigotica* Fort. is a traditional medicinal plant, which has anti-inflammatory, antioxidant, and antiviral properties. Despite the development and licensing of several cultivars in recent years, morphological similarity among cultivars complicates their identification. The genetic diversity within *I. indigotica* significantly impacts the biosynthesis of bioactive substances. To elucidate genetic relationships and evaluate bioactive compounds, *I. indigotica* cultivars were analyzed using SSR markers. A total of 109 alleles were identified across 29 cultivars at 20 SSR loci, exhibiting a genetic diversity with an average polymorphic information content (PIC) of 0.46. Phylogenetic, principal coordinate analysis (PCoA), and Bayesian clustering revealed that genetic relationships were largely independent of geographic origin, potentially due to regional transplantations. Notably, some cultivars with distinct leaf sizes showed clear genetic differentiation, highlighting their potential as candidates for quality evaluation. A fingerprint was successfully constructed using five SSR markers. These findings provide technical support for cultivar identification, quality evaluation, and intellectual property protection of *I. indigotica* cultivars.

## 1. Introduction

*Isatis indigotica* Fort. is a traditional Chinese medicine, which has antiviral, anti-inflammatory, immunomodulatory, and hypersensitivity-reducing properties [1,2,3,4]. The current research focuses are on the biological activity and bioactive components of *I. indigotica* [5,6,7]. However, studies on its genetic diversity and molecular markers remain limited, particularly using Simple Sequence Repeat (SSR) marker methods.

High-performance liquid chromatography (HPLC) has been widely applied to analyze bioactive components, such as indigotin, indirubin, uridine, progoitrin, epiprogoitrin, and gluconapin. It is valuable to establish relationships of bioactive compounds and diverse origins [8,9]. However, components analysis is not enough for classifying *I. indigotica* because of the influence of environmental factors or field management practices [10].

SSR markers are invaluable in genetic diversity studies, genome mapping, species identification, and population genetics for their abundance in genomes, codominant inheritance, and high polymorphism [11,12,13,14]. For *I. indigotica*, a germplasm resource system was important for standard planting aiming to obtain a steady yield and medicinal value. The development and application of SSR markers could clear up genetic background, and provide molecular methods for screening germplasm resources with target bioactive compounds or other valuable characteristics of *I. indigotica* cultivars [15,16].

With the advent of high-throughput sequencing technologies, genome-based SSR marker development has become feasible [17,18,19,20]. However, systematic research on SSR methods to classify *I. indigotica* is still lacking. This study aims to screen, validate SSR markers according to the *I. indigotica* genome, and analyze genetic diversity to establish a classification system using selected markers. These findings will serve as tools for germplasm screening and functional genomics research in *I. indigotica*. 

## 2. Materials and Methods

### 2.1. Plant Materials

**Figure 1:**
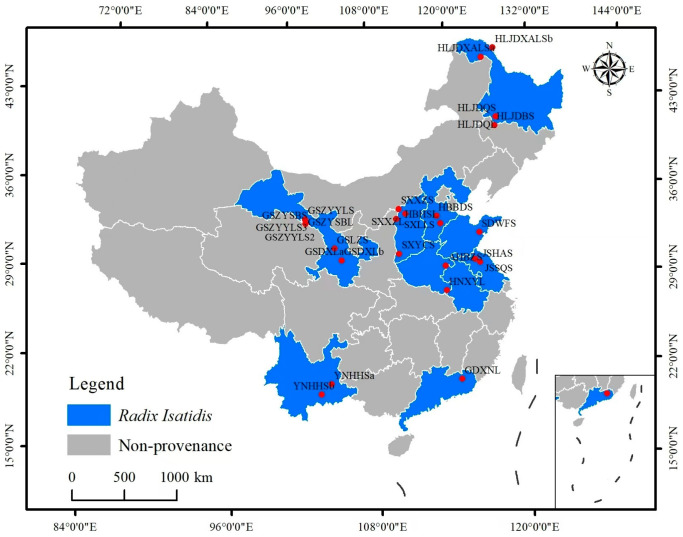
The origins distribution of 27 *I. indigotica* accessions.

A total of 29 seeds were used for SSR analysis. A total of 27 *I. indigotica* Fort. seeds were collected from major cultivation regions across China (Figure 1). In addition, standard seeds were obtained from National Institutes for Food and Drug Control. *Baphicacanthus cusia* (*Nees*) *Brem* seeds were obtained from National Institutes for Food and Drug Control, which are easily confused with *I. indigotica* and named BNIFDC.

**Figure 2:**
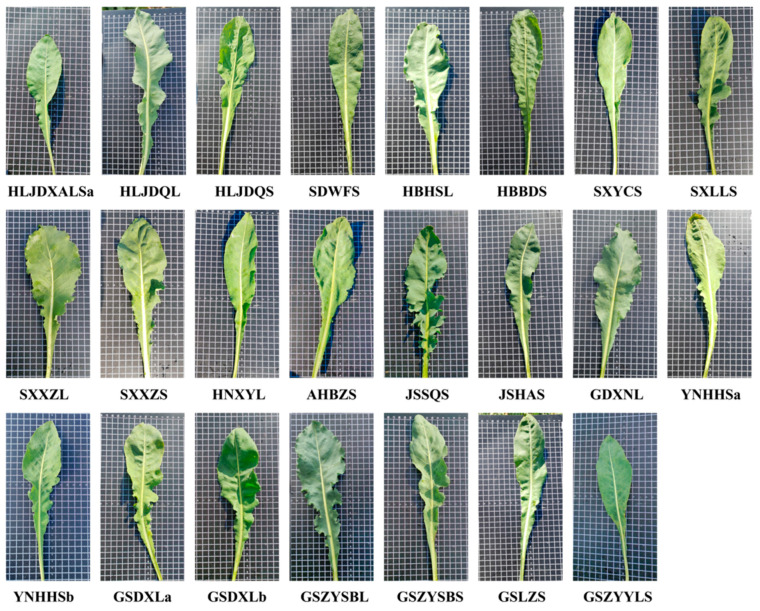
The leaf morphology of *I. indigotica* plants.

Detailed origins information of 27 accessions are provided in Table 1. The morphology of 23 *I. indigotica* plants are shown in Figure 2. The plant samples were kept in the Medicinal and Edible Homologous Plant Seed Laboratory. All 29 accessions were stored at −80 °C until DNA extraction.

### 2.2. DNA Extraction

Genomic DNA was extracted using a Rapid plant genomic DNA isolation kit (Sangon Biotech, Shanghai, China). DNA quality was measured with a NanoDrop-1000 spectrophotometer (NanoDrop Technologies, Wilmington, DE, USA), ensuring an OD260/OD280 ratio between 1.7 and 2.0. DNA samples were diluted to 20 ng/μL and stored at 4 °C for subsequent use. The Cetyl Trimethyl Ammonium Bromide (CTAB) method was also employed for DNA extraction [21,22]. A total of 100 mg of seeds was ground to a fine powder in liquid nitrogen, followed by incubation in prewarmed CTAB extraction buffer at 65 °C for 30 min. DNA was precipitated with cold isopropanol, washed with 70% ethanol, air-dried, and suspended in TE buffer. DNA concentration and quality were evaluated by a NanoDrop spectrophotometer. DNA was stored at −20 °C [23].

### 2.3. Genotyping with SSR Markers

Twenty SSR markers were selected based on the results of high-throughput sequencing. Briefly, the selected SSR markers were prioritized based on their high polymorphism levels (PIC values up to 0.84) and repeat length (>10 bp), ensuring reliable resolution in capillary electrophoresis. These markers were experimentally validated through preliminary screening to confirm amplification consistency and polymorphism using samples from HLJDXALSa, JSSQS, and YNHHSa. The selected SSR markers could differentiate all 29 accessions and construct the DNA fingerprinting system.

**Figure 3:**
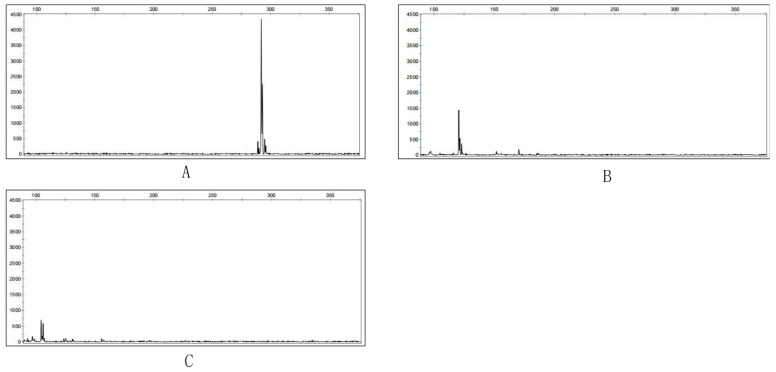
The capillary gel electrophoresis results of the amplification products of the BLG-P20 primer in the HLJDXALSa (**A**), JSSQS (**B**), and YNHHSa (**C**) samples.

Polymorphic primers were obtained (Figure 3). These polymorphic primers were selected for use in population diversity detection [24]. These markers produced clear and polymorphic bands. Fluorescently labeled primers with FAM, HEX, ROX, and NED were used for genotyping (Table 2). PCR reactions were carried out in a T100 thermal cycler (Bio-Rad Laboratories, Hercules, CA, USA) [25,26,27].

The 25 μL reaction mixture included 20 ng of DNA, 0.5 μL of 5 μM dNTP mix, 0.5 μL of 10 μM forward and reverse primers, 2.5 μL of buffer with MgCl_2_, and 1 U of Taq DNA polymerase. The PCR program consisted of denaturation at 95 °C for 5 min, followed by 10 cycles at 94 °C for 30 s, 60 °C for 30 s, and 72 °C for 30 s, followed by 30 cycles at 94 °C for 30 s, 55 °C for 30 s, and 72 °C for 30 s. The final extension was at 72 °C for 10 min. PCR products were analyzed by capillary electrophoresis using an ABI 3730xl sequencer (Thermo Fisher Scientific, Waltham, MA, USA). Genotypes were identified using a Bioelectrophoresis image analysis system (FR-980A, Furikeji, Shanghai, China). The base sizes of the bands were calculated with standard markers. The bands with the same base sizes were the same alleles in each pair of SSR markers [28,29].

### 2.4. Data Analysis

Genetic diversity parameters, including the number of alleles (Na), effective number of alleles (Ne), observed heterozygosity (Ho), expected heterozygosity (He), Shannon’s diversity index (I), and polymorphic information content (PIC), were calculated using software PopGen version 1.32 (North Carolina State University, Raleigh, NC, USA) [30]. Principal coordinate analysis (PCoA) was performed using GenAlEx version 6, and Bayesian clustering analysis was conducted with STRUCTURE version 5.3.1 to assess population structure (Stanford University, San Francisco, CA, USA) [31].

## 3. Results

### 3.1. Genetic Diversity

A total of 109 alleles (Na) were identified across 20 SSR loci among the 29 accessions, with the number of alleles ranging from 2 to 11. The amplified fragments varied from 105 bp for BLG-P1 to 315 bp for BLG-P19. The locus BLG-P13 exhibited the highest number of alleles (11), while BLG-P14, BLG-P6, and BLG-P9 had the lowest (2–3). The average effective number of alleles (Ne) was 2.33 (Table 3).

A high proportion of homozygotes (Ho > 0.5) was observed at 12 SSR loci, with BLG-P1 and BLG-P16 exhibiting the highest values. The polymorphism information content (PIC) ranged from 0.16 to 0.84, with an average of 0.47. BLG-P13 had the highest PIC value, indicating greater genetic variation, whereas BLG-P1 had the lowest. Shannon’s diversity index (I) ranged from 0.42 to 2.11, with an average of 0.98, reflecting considerable genetic diversity among the cultivars.

### 3.2. Genetic Differentiation

**Figure 4:**
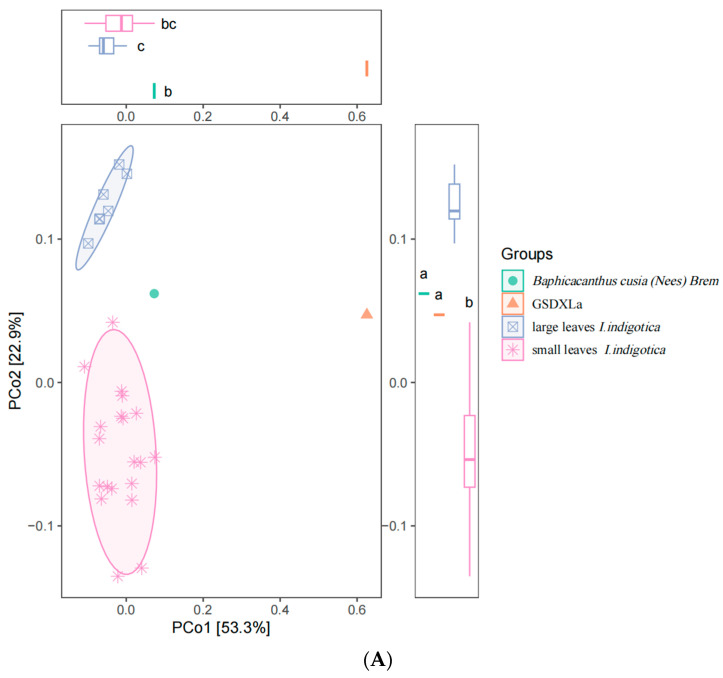

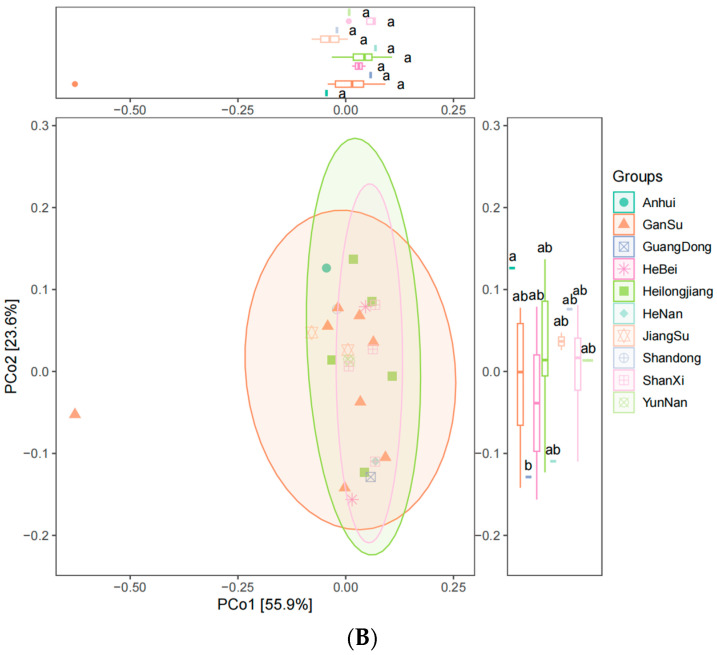
The principal component analysis (PCA) score plot depicting the distribution of 29 *Isatis indigotica* Fort cultivars for the first two principal component (PCo1 and PCo2). Individuals are color-coded with large leaves or small leaves (**A**) and geographical origin (**B**). Significant differences (*p* ≤ 0.05) are indicate by different letter.

**Figure 5:**
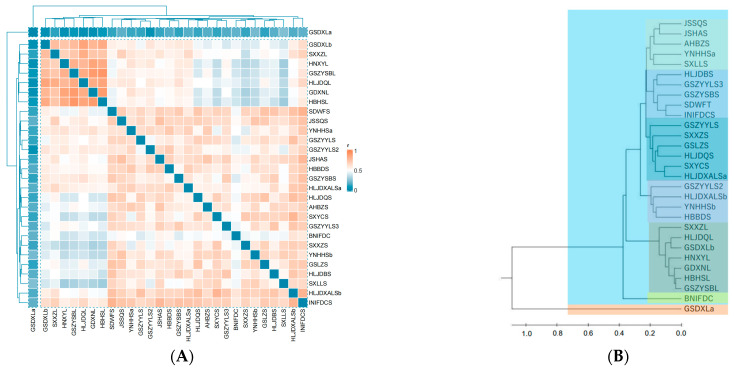

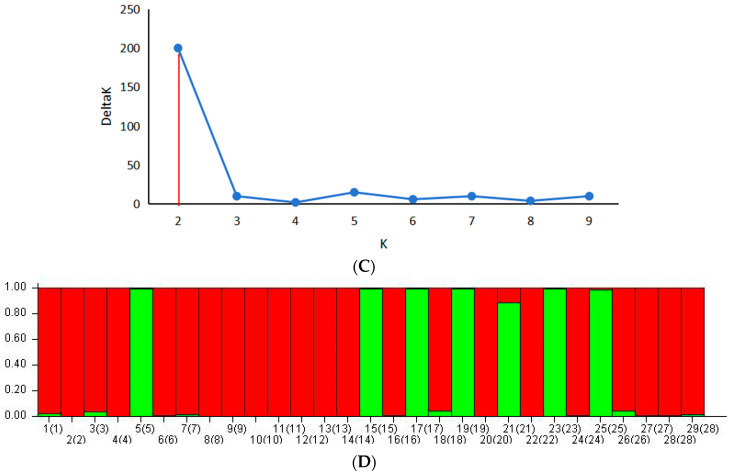
(**A**) A heat map of genetic distance calculated by popgene32 UPGMA; (**B**) a clustering tree constructed by genetic distance, each color represent one clusters; (**C**) a graphical depiction of the relationship between K and ∆k; (**D**) Bayesian clustering at K = 2, each color represents one group.

Principal coordinate analysis (Figure 4A) revealed that the genetic relationships among *I. indigotica* cultivars were independent of leaf morphology (Figure 4A). Distinct genetic differences were observed among cultivars with small and large leaves. Interestingly, *Baphicacanthus cusia (Nees)* Brem showed significant divergence from *I. indigotica*, which is usually confused with *I. indigotica.* GSDXLa was the most genetically distinct cultivar. Principal coordinate analysis (Figure 4B) revealed that the phylogenetic relationships among different cultivars exhibit no significant correlation with the geographical origins of the sampled specimens (Figure 4B). Clustering analysis using the neighbor-joining method confirmed these findings and revealed six genetic subclasses (Figure 5B).

The genetic distance ranged from 0.039 to 0.927, with an average of 0.545 among the 29 accessions. GSDXLa and GDXNL have the closest genetic distance (0.039), followed by GSDXLa and HNXYL (0.040). In contrast, GSZYSBL and HBHSL have the farthest genetic distance (0.93), followed by GSZYSBL and GDXNL (0.91). Standard materials INIFDCS and GSDXLa are the closest (0.18) (Figure 5A).

A dendrogram was created by an unweighted pair-group method with the arithmetic mean (UPGMA) method. From the dendrogram, GSDXLa of the 29 *I. indigotica* was independent from other materials, and the other materials could be roughly divided into six subpopulations (Figure 5B).

From the genetic distance, GSDXLa of the 29 accessions was independent from other materials, and the other materials could be roughly divided into six subclasses.

The K value was important for clarifying the genetic value among samples to help identify genetic stratification within the population and showing which individuals belong to similar genetic backgrounds [32]. The K value change is shown in Figure 5C; delta K was the largest when K was two. All 29 accessions tested can be divided into four subpopulations based on K = 2 (Figure 5C). Subpopulations I, with the same pure red color, were composed of 16 accessions, which were JSSQS, SDWFS, JSHAS, GSLZS, AHBZS, YNHHSb, HLJDBS, SXYCS, SXLLS, HLJDXALSb, HBBDS, HLJDQS, SXXZS, GSDXLa, GSZYYLS3, and BNIFDC. Subpopulations II, with the same pure green color, had six accessions, which were NXYL, HBHSL, GSZYSBL, HLJDQL, GDXNL, and GSDXLb. The individuals in subpopulations I and II had a pure lineage. Subpopulations III, with a mixed color closer to subpopulations I, were composed of six accessions, which were HLJDXALSa, NHHSa, GSZYYLS, GSZYSBS, GSZYYLS2, and INIFDCS. Subpopulations IV were closer to subpopulations II and only had SXXZL.

### 3.3. Unique Alleles

A total of 29 unique alleles were obtained in the 20 SSR loci, which means that the alleles can be used as characters of one cultivar (Table 4). The primers, such as BLG-P9, BLG-P11, BLG-P14, BLG-P15, BLG-P17, and BLG-P18, had no unique alleles (Table 2). The remaining 14 SSR primers had one or two unique alleles. SDWFS, GSZYYLS, AHBZS, SXLLS, HLJDXALSb, HBHSL, GSZYSBL, HLJDQL, GDXNL, and GSDXLb had no unique alleles. BNIFDC, which is distinct from *I. indigotica*, had five unique alleles aligning with its distinct genetic characteristics.

### 3.4. Fingerprinting of Cultivars

**Figure 6:**
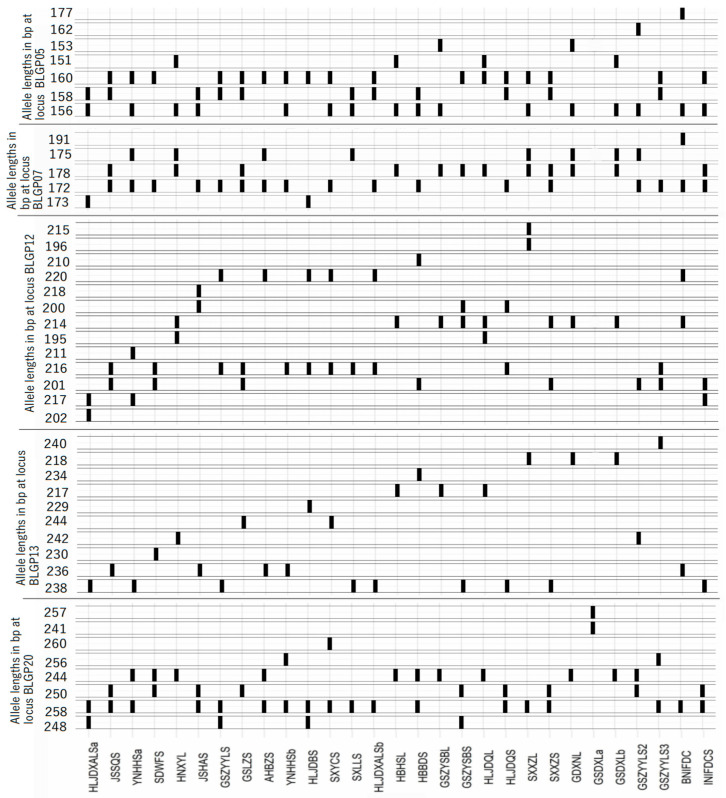
Fingerprints of the 29 cultivars based on five SSR loci.

To distinguish and identify *I. indigotica* cultivars, a DNA fingerprint was constructed using five SSR markers: BLG-P13, BLG-P12, BLG-P5, BLG-P20, and BLG-P7 (Figure 6). The combined use of these markers successfully differentiated all 29 cultivars. BLG-P12 and BLG-P13 exhibited the highest PIC values (0.79 and 0.84, respectively), underscoring their utility in genetic identification. BLG-P13, BLG-P12, BLG-P5, BLG-P20, and BLG-P7 can be used as SSR markers for differentiating *I. indigotica*. The PCR products were analyzed by capillary electrophoresis. The accessions had the same bands, which means the same original of the cultivars (Figure 6). It was meaningful for seed traceability and breeding.

## 4. Discussion

Genetic diversity plays an important role in a species’ morphological characteristics and biological activities, and is essential for its evolution [33]. In plant breeding, genetic diversity is a valuable resource for developing new cultivars [34,35]. Previous research on the genetic diversity of *I. indigotica* mainly focused on the use of ISSR (Inter-Simple Sequence Repeat) markers [36]. Compared with an ISSR, an SSR sequence has more polymorphism and can distinguish heterozygotes and homozygotes.

In this study, the 29 accessions displayed high genetic diversity, with Shannon’s diversity index (I = 0.98) and polymorphic information content (PIC = 0.465), comparable to those reported for jute (*Corchorus* spp.), grass pea (*Lathyrus sativus* L.), and chrysanthemum germplasm using SSR markers [37,38,39]. The results of principal coordinate analysis (PCoA) using SSR markers divided *I. indigotica* cultivars into two phenotypic groups based on leaf size: large-leaf and small-leaf cultivars. Generally, cultivars with large leaves typically have more lateral roots, whereas cultivars with small leaves have fewer lateral roots. Long and straight roots are good for processing into decoction pieces, meaning that small-leaf cultivars were more popular. This differentiation suggests that the SSR marker system developed in this study has potential value for seed selection and seed quality control for the *I. indigotica* industry.

Hybridization, whether natural or artificial, is a vital strategy in generating new plant cultivars [40,41]. The establishment of homozygous genotypes is a prerequisite for stable inheritance in crossbreeding programs [42,43]. Among the 29 accessions analyzed, 22 showed homozygous genotypes, which are valuable resources for crossbreeding to get desirable traits.

Geographic distance and selection pressures often influence genetic differentiation among populations [44]. However, the 29 accessions analyzed in this study, originating from 10 provinces in China, did not exhibit clear geographic clustering. This result may stem from extensive transplantations, which have diluted regional genetic distinctions.

Morphological traits alone are often insufficient to differentiate *I. indigotica* cultivars due to their phenotypic plasticity, which is influenced by both genetic and environmental factors [45]. On the one hand, the main difference among *I. indigotica* cultivars is functional composition content, which is difficult to ascertain because the active ingredients need to be determined was in the root [46]. On the other hand, the phenotype of plants is controlled by both genetic and environmental factors. Cultivars planted in different environments may have different morphological traits, which may result in misidentification and confusion [47]. Molecular fingerprinting using SSR markers can avoid these limitations, enabling accurate identification [48]. In this study, there were 109 alleles identified at 20 SSR loci, which suggests genetics different markedly among the cultivars. An SSR marker system was not reported in previous studies for *I. indigotica*. In this study, 29 unique alleles were identified and constructed a DNA fingerprint using five SSR markers (BLG-P13, BLG-P12, BLG-P5, BLG-P20, and BLG-P7). It is meaningful for quality control, intellectual property protection, and authenticity verification.

## 5. Conclusions

This study constructed an SSR method system for assessing the genetic diversity and population structure of *I. indigotica*. The results demonstrated high genetic diversity (Shannon’s diversity index, I = 0.98; polymorphic information content, PIC = 0.465) and revealed significant genetic differentiation among cultivars. Phylogenetic and PCoA analyses indicated that genetic relationships were independent of geographic origin, which may be due to extensive regional transplantations.

The developed SSR marker-based fingerprinting system successfully distinguished all 29 cultivars, providing a reliable tool for genetic identification, quality evaluation, and intellectual property protection. Five highly informative SSR markers (BLG-P13, BLG-P12, BLG-P5, BLG-P20, and BLG-P7) were identified as the core loci for cultivar differentiation. These findings not only enhance our understanding of *I. indigotica*’s genetic diversity but also offer practical applications for seed selection and breeding programs.

In conclusion, this study constructed an SSR marker-based fingerprinting system for *I. indigotica*, which was meaningful for authenticating, molecular breeding, quality control, and conservation, which will prompt *I. indigotica* industry development and utilization.

## Figures and Tables

**Table 1:** The origins details of the 27 analyzed *I. indigotica* accessions.

Number	Cultivar	Origin	Leave Shape	Number	Cultivar	Origin	Leave Shape
1	HLJDXALSa	Heilongjiang, Daxinganling	Small	15	HBHSL	HeBei, HengShui	Large
2	JSSQS	JiangSu, SuQian	Small	16	HBBDS	HeBei, BaoDing	Small
3	YNHHSa	YunNan, HongHe	Small	17	GSZYSBL	GanSu, ZhangYe	Large
4	SDWFS	Shandong, Weifang	Small	18	GSZYSBS	GanSu, ZhangYe	Small
5	HNXYL	HeNan, XinYang	Large	19	HLJDQL	HeiLongJiang, DaQing	Large
6	JSHAS	Jiangsu, Huaian	Small	20	HLJDQS	HeiLongJiang, DaQing	Small
7	GSZYYLS	Gansu, Minle	Small	21	SXXZL	ShanXi, XinZhou	Large
8	GSLZS	Gansu, Lanzhou	Small	22	SXXZS	ShanXi, XinZhou	Small
9	AHBZS	Anhui, Bozhou	Small	23	GDXNL	GuangDong, XingNing	Large
10	YNHHSb	YunNan, HongHe	Small	24	GSDXLa	GanSu, DingXi	Large
11	HLJDBS	Heilongjiang, Duerbote	Small	25	GSDXLb	GanSu, DingXi	Large
12	SXYCS	ShanXi, YunCheng	Small	26	GSZYYLS2	Gansu, Minle	Small
13	SXLLS	ShanXi, LvLiang	Small	27	GSZYYLS3	Gansu, Minle	Small
14	HLJDXALSb	Heilongjiang, Daxinganling	Small				

**Table 2:** Information on the 20 polymorphic SSR markers.

Locus	Repeat Motif	Forward Primer (5′~3′)	Reverse Primer (5′~3′)	Size	Tm	Fluorescent Labeling
BLG-P1	(GA)10	TCTCTTGATTCTTTTTGACGGA	TCGTTTCCTGTTCCCTTTTG	109	51	FAM
BLG-P2	(TC)10	GTGTTTGTGTTTCCCCCATC	GAAAAACGGTGCCACAATCT	121	53	FAM
BLG-P3	(GA)12	CGAATTTACCACGAACCGAT	GAAAACGGTGGCATGTCTCT	136	53	FAM
BLG-P4	(TC)10	CAAGCACAAGTGGTCCAAAA	GCTTGGTTTTCAACATGAGG	151	52	FAM
BLG-P5	(TG)12	AGAAGGCTGCACCAAGTGTT	GAGGAAGGATCCAAATGCAA	157	54	FAM
BLG-P6	(GA)11	CTTCCCATTTAGCGAACCAA	CTTCCGGTTCGATTTTTCAA	165	51	HEX
BLG-P7	(GTT)9	TCGTTCGGTTATGACGGCTCTT	CGTAAGGTCCAATGGCGAATAT	176	55	HEX
BLG-P8	(ACCAAT)7	CTCCAAGACCATCTTCCCAA	TGGGAAAAAGACAGGCAATC	180	53	HEX
BLG-P9	(AG)11	ACTCTCAGGGCAGCGACAGAAA	TCTCCCACCACCACCACAAATA	192	58	HEX
BLG-P10	(TC)13	TTCGATTATTGGGCGAAGTT	TAGCCACACCGAGATCAAGA	193	53	HEX
BLG-P11	(TC)10	TAAACCGTCGCAACAGAGAC	ACCTGCCATTGCCTAACAAG	201	55	ROX
BLG-P12	(TC)15	ATTTCGGTGCATTGCTTTCT	TAACTTCTTCGGTCTTGCCG	204	52	ROX
BLG-P13	(AT)16	CACCATTAATAGGAATGTGGCA	TTTAATGCATGGTTGGCATC	210	51	ROX
BLG-P14	(TG)12	TGGAGCAAGAAGAGAGGTTAGG	TTTGAAGCTCTGCAGGGAAAGT	212	55	ROX
BLG-P15	(AG)15	TGAGCATGCGAATCAAACTC	CGAATTGGGGAGATATTGGA	235	52	ROX
BLG-P16	(AG)11	GACATTTCCACCAGCAAGGT	AAGTGCTAGTTGGAAGCCGA	248	55	NED
BLG-P17	(AG)12	CAAACCACCACCGGACCACTAT	GCCTCTCCATCCTCGTCGTATT	253	58	NED
BLG-P18	(CA)15	TCCCCTTCTTTCTTCTATTGC	TCTCCGCCATAGATTTCTGC	257	53	NED
BLG-P19	(GA)13	TATGTAGCCATCCCTGCCTC	ATGGCGTCAATGACATACCA	274	53	NED
BLG-P20	(TC)11	TGGGAAGGAAGAAGAAGCAA	TGACGACAACGACTTCAACA	279	52	NED

**Table 3:** Results of the genetic analysis of the 20 SSR loci.

Locus	Sample Size	Na	Ne	Ho	He	I	PIC
BLG-P1	(GA)10	5	1.20	0.86	0.14	0.42	0.16
BLG-P2	(TC)10	4	1.52	0.66	0.34	0.63	0.30
BLG-P3	(GA)12	7	2.53	0.34	0.66	1.21	0.55
BLG-P4	(TC)10	6	2.60	0.38	0.62	1.17	0.55
BLG-P5	(TG)12	7	3.73	0.143	0.86	1.50	0.69
BLG-P6	(GA)11	3	2.06	0.36	0.64	0.77	0.40
BLG-P7	(GTT)9	5	2.85	0.57	0.43	1.23	0.59
BLG-P8	(ACCAAT)7	5	2.65	0.52	0.48	1.13	0.56
BLG-P9	(AG)11	3	1.82	0.68	0.32	0.76	0.39
BLG-P10	(TC)13	5	1.40	0.71	0.29	0.64	0.27
BLG-P11	(TC)10	4	2.13	0.64	0.36	0.99	0.48
BLG-P12	(TC)15	10	5.39	0.29	0.71	1.87	0.79
BLG-P13	(AT)16	11	7	0.25	0.75	2.11	0.84
BLG-P14	(TG)12	2	1.67	0.74	0.26	0.59	0.32
BLG-P15	(AG)15	5	3.97	0.26	0.74	1.47	0.71
BLG-P16	(AG)11	5	1.25	0.86	0.14	0.48	0.19
BLG-P17	(AG)12	4	1.69	0.50	0.50	0.79	0.37
BLG-P18	(CA)15	4	2.55	0.52	0.48	1.10	0.55
BLG-P19	(GA)13	7	3.53	0.69	0.31	1.55	0.69
BLG-P20	(TC)11	7	3.58	0.38	0.62	1.47	0.68
Mean		5	2.33	0.52	0.48	0.98	0.465

Note: Na = Observed number of alleles; Ne = Effective number of alleles; Ho = Observed heterozygosity; He = Expected heterozygosity; I = Shannon’s Information index; PIC = polymorphic information content.

**Table 4:** Genetic analysis of the 29 SSR unique alleles of *I. indigotica*.

Locus	Unique Allele Lengths in bp (Locus)	Number of Unique Alleles
HLJDXALSa	202 (BLGP12); 288 (BLGP16)	2
JSSQS	165 (BLGP6)	1
YNHHSa	212 (BLGP12)	1
HNXYL	179 (BLGP4)	1
JSHAS	248 (BLGP13); 121 (BLGP2)	2
GSLZS	316 (BLGP19)	1
YNHHSb	119 (BLGP1)	1
HLJDBS	298 (BLGP16)	1
SXYCS	260 (BLGP20)	1
HBBDS	210 (BLGP12); 234 (BLGP13); 296 (BLGP19)	3
GSZYSBS	117,124 (BLGP1)	1
HLJDQS	128 (BLGP3)	1
SXXZL	196 (BLGP12); 252 (BLGP13)	2
SXXZS	284 (BLGP19)	1
GSDXLa	113 (BLGP2); 153 (BLGP4); 179 (BLGP8); 240 (BLGP20)	4
GSZYYLS2	161 (BLGP5)	1
GSZYYLS3	140 (BLGP3)	1
BNIFDC	177 (BLGP5); 191 (BLGP7); 201 (BLGP10); 209 (BLGP10); 294 (BLGP16)	5
Total		29

## Data Availability

Data are contained within the article.
